# Развитие саркоидоза после успешного лечения болезни Иценко–Кушинга

**DOI:** 10.14341/probl13203

**Published:** 2024-01-24

**Authors:** П. А. Захарова, И. А. Иловайская, С. А. Терпигорев, И. В. Комердус, А. Ю. Луговская

**Affiliations:** Московский областной научно-исследовательский клинический институт им. М.Ф. Владимирского; Московский областной научно-исследовательский клинический институт им. М.Ф. Владимирского; Национальный медико-хирургический центр им. Н.И. Пирогова; Московский областной научно-исследовательский клинический институт им. М.Ф. Владимирского; Московский областной научно-исследовательский клинический институт им. М.Ф. Владимирского

**Keywords:** саркоидоз, гиперкортицизм, болезнь Иценко–Кушинга, глюкокортикостероиды, синдром Лефгрена, кортикотропинома

## Abstract

Болезнь Иценко–Кушинга — это редкое тяжелое нейроэндокринное заболевание, обусловленное хронической гиперпродукцией адренокортикотропного гормона опухолью гипофиза. Высокие концентрации уровня кортизола в крови при эндогенном гиперкортицизме оказывают иммуносупрессивное и противовоспалительное действие, так же как и терапия системными глюкокортикостероидами. Это может способствовать снижению активности имеющихся у пациента сопутствующих аутоиммунных воспалительных заболеваний. С другой стороны, снижение уровня кортизола на фоне лечения болезни Иценко–Кушинга может быть ассоциировано с реактивацией иммунной системы, что увеличивает риск рецидива или дебюта различных аутоиммунных заболеваний. Мы приводим собственный клинический случай, демонстрирующий сложности диагностики эндогенного гиперкортицизма у пациентки молодого возраста и последующее развитие саркоидоза, возникшего после успешного оперативного лечения болезни Иценко–Кушинга.

## Abstract (en)

Cushing’s disease is a rare severe neuroendocrine disorder caused by chronic overproduction of adrenocorticotropic hormone by a pituitary tumor. Supraphysiological concentrations of cortisol in endogenous hypercortisolism have an immunosuppressive and anti-inflammatory effect similar to therapy with systemic glucocorticosteroids. This may reduce the activity of the patient’s concomitant autoimmune inflammatory diseases. On the other hand, a decrease in cortisol levels during treatment for Cushing’s disease may be associated with a reactivation of the immune system that pose a risk of onset or recurrence of an autoimmune disorder. We present our own clinical case demonstrating the development of sarcoidosis after surgical treatment of Cushing’s disease.

## АКТУАЛЬНОСТЬ

Болезнь Иценко–Кушинга — это редкое тяжелое нейроэндокринное заболевание, обусловленное хронической гиперпродукцией адренокортикотропного гормона (АКТГ) опухолью гипофиза (кортикотропиномой). Постоянная супрафизиологическая секреция АКТГ приводит к избыточной выработке кортизола корой надпочечников и развитию эндогенного гиперкортицизма [1][2]. Среди характерных симптомов гиперкортицизма отмечают ожирение, артериальную гипертензию, нарушения углеводного обмена и снижение минеральной плотности кости [3]. Однако сочетание ожирения, артериальной гипертензии и нарушений углеводного обмена встречается с частотой до 20–30% в общей популяции в рамках метаболического синдрома [4–7]. Мировое сообщество не рекомендует проводить скрининг эндогенного гиперкортицизма у всех лиц с ожирением и сахарным диабетом ввиду широкой распространенности этих заболеваний. Тем не менее частота метаболического синдрома прямо коррелирует с возрастом, и чем моложе пациент с метаболическим синдромом, тем более оправдано исключение эндогенного гиперкортицизма.

Высокие концентрации кортизола при эндогенном гиперкортицизме оказывают иммуносупрессивное и противовоспалительное действия, так же как и терапия системными глюкокортикостероидами. Это может способствовать снижению активности имеющихся у пациента сопутствующих аутоиммунных воспалительных заболеваний (например — ревматоидного артрита). С другой стороны, снижение уровня кортизола на фоне лечения болезни Иценко–Кушинга может быть ассоциировано с реактивацией иммунной системы, что увеличивает риск рецидива или дебюта аутоиммунного заболевания. В медицинской литературе имеются немногочисленные описания случаев возникновения или обострения ревматоидного артрита [8], атопического дерматита, ­аутоиммунного тиреоидита [9], серонегативного артрита, васкулита сетчатки глаз [10], пузырчатки [11], а также саркоидоза [12] после успешного лечения эндогенного гиперкортицизма. Мы приводим собственный клинический случай, демонстрирующий сложности диагностики эндогенного гиперкортицизма у пациентки молодого возраста и развитие саркоидоза, возникшего после оперативного лечения болезни Иценко–Кушинга.

## ОПИСАНИЕ СЛУЧАЯ

Пациентка М. считала себя здоровой до 28 лет, в этом возрасте масса тела была 80–85 кг при росте 175 см, индекс массы тела (ИМТ) 27,1 кг/м², АД 120–130/70–80 мм рт.ст., менструации начались с 12 лет, цикл был регулярным. В возрасте 20 лет и 24 года — беременности, наступившие самостоятельно, закончившиеся родами в срок через естественные родовые пути без осложнений.

В 28 лет во время третьей беременности прибавила в весе 40 кг, отмечались повышение АД до 150/120 мм рт.ст., избыточный рост волос на лице и спине, появление стрий на животе и молочных железах. Во время беременности принимала эналаприл 5 мг. К концу беременности масса тела составила 120 кг при росте 175 см (ИМТ 39 кг/м²). Беременность закончилась срочными родами здорового ребенка. После родов вес оставался прежним, сохранялось повышение АД до 150/100 мм рт.ст., принимала периодически эналаприл 5 мг, к врачам не обращалась.

В 30 лет пациентка отметила, что менструальный цикл после беременности так и не восстановился, масса тела увеличилась еще на 5 кг (ИМТ 40,8 кг/м2), беспокоил рецидивирующий фурункулез. С вышеперечисленными жалобами обратилась к терапевту, однако гормонального обследования не проводилось. Был поставлен диагноз ожирения 3-й степени, все изменения интерпретированы как последствия ожирения, рекомендована диетотерапия — без какого-либо эффекта. Назначен сибутрамин в дозе 15 мг, в течение 3 мес на фоне приема похудела на 15 кг (масса тела 110 кг), однако в связи с повышением АД препарат был отменен, после отмены препарата в течение 1 года постепенно масса тела увеличилась до 125–130 кг.

В 32 года обратилась к эндокринологу по месту жительства. На основании сочетания ожирения, артериальной гипертензии, нарушений менструального цикла, гирсутизма у пациентки молодого возраста заподозрен эндогенный гиперкортицизм. Кроме того, было показано гормональное обследование для исключения вторичного характера артериальной гипертензии и уточнения эндокринных причин нарушений менструального цикла. При гормональном обследовании исключены нарушения функции щитовидной железы, гиперальдостеронизм, акромегалия, гиперпролактинемия и феохромоцитома; выявлено значимое повышение экскреции свободного кортизола в суточной моче — 635,2 нмоль/сут (100–379), что явилось основанием для более тщательного обследования.

В 33 года госпитализирована в эндокринологический стационар, при осмотре вес 130 кг, рост 175 кг, ИМТ 42,4 кг/м², подкожно-жировая клетчатка развита избыточно, перераспределена по центральному типу: окружность талии (ОТ) 144 см, окружность бедер (ОБ) 124 см, ОТ/ОБ 1,16. На кожных покровах обращало на себя внимание наличие келлоидных рубцов на месте фурункулов; синюшные стрии на передней поверхности живота, молочных желез, шириной 7–10 мм, длиной до 20 см; избыточный рост волос терминального типа над верхней губой, подбородка, щек, белой линии живота, нижних и верхних конечностей, крестца, гирсутное число по шкале Ферримана–Галвея 14 баллов (умеренно выраженный гирсутизм). АД 180/120 мм рт.ст., ЧСС 82 уд/мин, в остальном органы кровообращения без отклонений. Осмотр органов дыхания, пищеварения и мочеполовой системы без особенностей.

Согласно алгоритму диагностики эндогенного гиперкортицизма, на первом этапе диагностического поиска для исключения/подтверждения гиперсекреции кортизола проводят 1 или 2 исследования из 3 возможных: исследование суточной экскреции свободного кортизола с мочой, малый дексаметазоновый тест, определение уровня свободного кортизола в слюне вечером. У пациентки было выявлено повышение суточной экскреции свободного кортизола с мочой и отсутствие физиологического подавления продукции кортизола после приема 1 мг дексаметазона (табл. 1), что подтвердило наличие эндогенного гиперкортицизма и явилось основанием для продолжения обследования.

**Table:** Таблица 1. Гормональное обследование на наличие эндогенного гиперкортицизма и его дифференциальная диагностика

Показатель	Значение	Референсные значения
Кортизол крови в ходе ночной подавляющей пробы с 1 мг дексаметазона	726 нмоль/л	менее 50 нмоль/л
Свободный кортизол в суточной моче	335,9 нмоль/л/сут	80–250
АКТГ утром	8,1 пмоль/л	3,3–11
АКТГ вечер	8,0 пмоль/л	3,3–11
Большая дексаметазоновая проба с 8 мг
Кортизол исходно	486 нмоль/л
Кортизол после подавления	82 нмоль/л
Процент подавления	83,1%

На следующем этапе диагностического поиска проводится уточнение причин гиперкортицизма, наиболее частой причиной которого является АКТГ-продуцирующая опухоль гипофиза (кортикотропинома), однако могут быть случаи и кортизол-продуцирующих опухолей надпочечников или эктопической продукции АКТГ нейроэндокринными опухолями внегипофизарной локализации. Исследован суточный ритм АКТГ (физиологический ритм отсутствовал) и проведена большая дексаметазоновая проба (табл. 1), которые подтвердили наличие АКТГ-­зависимого гиперкортицизма. По данным МРТ гипоталамо-гипофизарной области (на аппарате со сверхвысоким напряжением магнитного поля 3,0 Ел) без контрастирования: в проекции увеличенного в размерах турецкого седла и супраселлярной цистерны срединно выявлено объемное образование округлой формы с четкими ровными контурами, размерами 17×15×12 мм, неоднородной структуры: преимущественно гиперинтенсивное на Т1-ВИ (взвешенных изображениях) и Т2-ВИ с участками гипоинтенсивного сигнала (рис. 1). Для исключения участков кровоизлияний была проведена КТ головного мозга с контрастированием: в полости турецкого седла определяется округлое объемное образование с супраселлярным распространением, с четкими ровными контурами, размерами 17×12×15 мм; структура образования неоднородная; в нижних отделах плотность образования при нативном сканировании — 53 ед. Н, в верхних — 39 ед. Н; после внутривенного контрастного усиления отмечалось накопление контрастного препарата только нижними отделами образования; КТ-данных за кровоизлияние в опухоль не получено.

**Figure:**
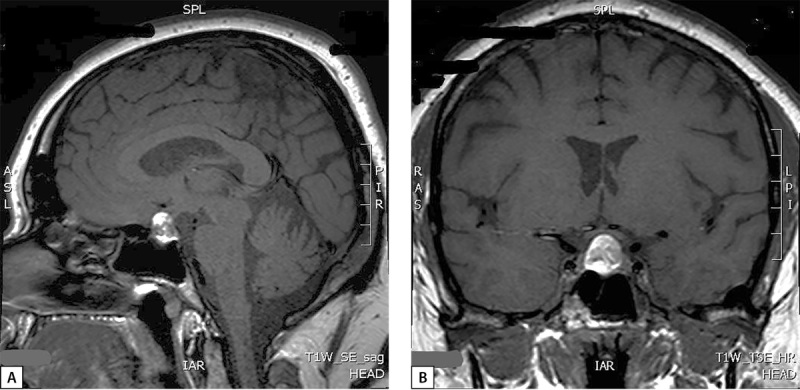
Рисунок 1. Снимки МРТ гипофиза в сагиттальной (А) и фронтальной (В) проекциях пациентки М., 33 года. Визуализируется супра-эндоселлярная аденома гипофиза.

Первой линией лечения при болезни Иценко–Кушинга является нейрохирургическое удаление опухоли гипофиза [1], в связи с этим нашей пациентке была проведена эндоскопическая трансназальная аденомэктомия. В послеоперационном периоде отмечались снижение артериального давления до 100–110/60–70 мм рт.ст. без приема антигипертензивной терапии, мышечная слабость, отсутствие аппетита. Уровень кортизола крови утром составил 64 нмоль/л (119–618), что соответствовало вторичной надпочечниковой недостаточности, развитие которой является благоприятным прогностическим маркером радикального удаления кортикотропиномы [1][2]. По данным гистологического исследования: базофильная аденома гипофиза с инфильтрацией прилежащей глиальной ткани. По данным иммуногистохимического исследования удаленной опухолевой ткани было подтверждено наличие кортикотропиномы: отмечено выраженное диффузное окрашивание цитоплазмы клеток антителами к АКТГ (оценка экспрессии Ki-67 в данном случае не проводилась). Для компенсации надпочечниковой недостаточности пациентке был назначен гидрокортизон в заместительной дозе 15 мг/сут (в 2 приема — 10 мг утром и 5 мг во второй половине дня). На фоне приема гидрокортизона самочувствие улучшилось, аппетит нормализовался, слабость прошла, АД стабилизировалось на уровне 120/80 мм рт.ст. В течение последующих 3 мес наблюдался регресс симптомов гиперкортицизма: снизилась масса тела на 15 кг, стало более равномерным перераспределение подкожно-жировой клетчатки, уменьшились в размерах и побледнели стрии, отметила менее интенсивный рост волос на лице и теле, восстановился менструальный цикл. Пациентка продолжала терапию гидрокортизона в дозе 15 мг/сут.

Через 4 мес после хирургического лечения у пациентки повысилась температура тела до 37,6–37,8°С, стали беспокоить артралгии, появились общая и мышечная слабость, тошнота. Через несколько дней после повышения температуры тела появилась типичная картина узловатой эритемы на коже голеней, по поводу чего пациентка была госпитализирована в терапевтический стационар. При первичном осмотре рост 175 см, масса тела 115 кг, ИМТ 38 кг/м², окружность талии 130 см, окружность бедер 131 см, ОТ/ОБ 1; кожные покровы обычной окраски, в левой и правой подключичных областях плотные образования темно-коричневого цвета диаметром 0,5–1 см, на передней поверхности живота стрии светло-розового цвета шириной около 0,5 см. Аускультация легких — дыхание с жестким оттенком, хрипы не выслушивались. Осмотр органов кровообращения, пищеварения и мочеполовой системы — без особенностей.

При КТ органов грудной клетки (рис. 2, 3) выявлены множественные мелкоочаговые затемнения в легочной паренхиме лимфогенного распределения, немногочисленные ретикулярные затемнения, уплотнение перибронховаскулярного интерстиция и увеличение лимфоузлов средостения и корней легких, т.е. симптомокомплекс, высокоспецифичный для острой формы саркоидоза. Лабораторные данные подтвердили активность системного воспаления: скорость оседания эритроцитов (СОЭ) 42 мм/ч (0–20), уровень С-реактивного белка (СРБ) был повышен до 7,23 мг/л (норма 0,01–4,99). Уровень кальция крови был в пределах нормальных значений. Проводилась дифференциальная диагностика с туберкулезом органов дыхания и лимфопролиферативными заболеваниями, результаты обследования пациентки и оценка течения заболевания позволили исключить эти заболевания.

**Figure:**
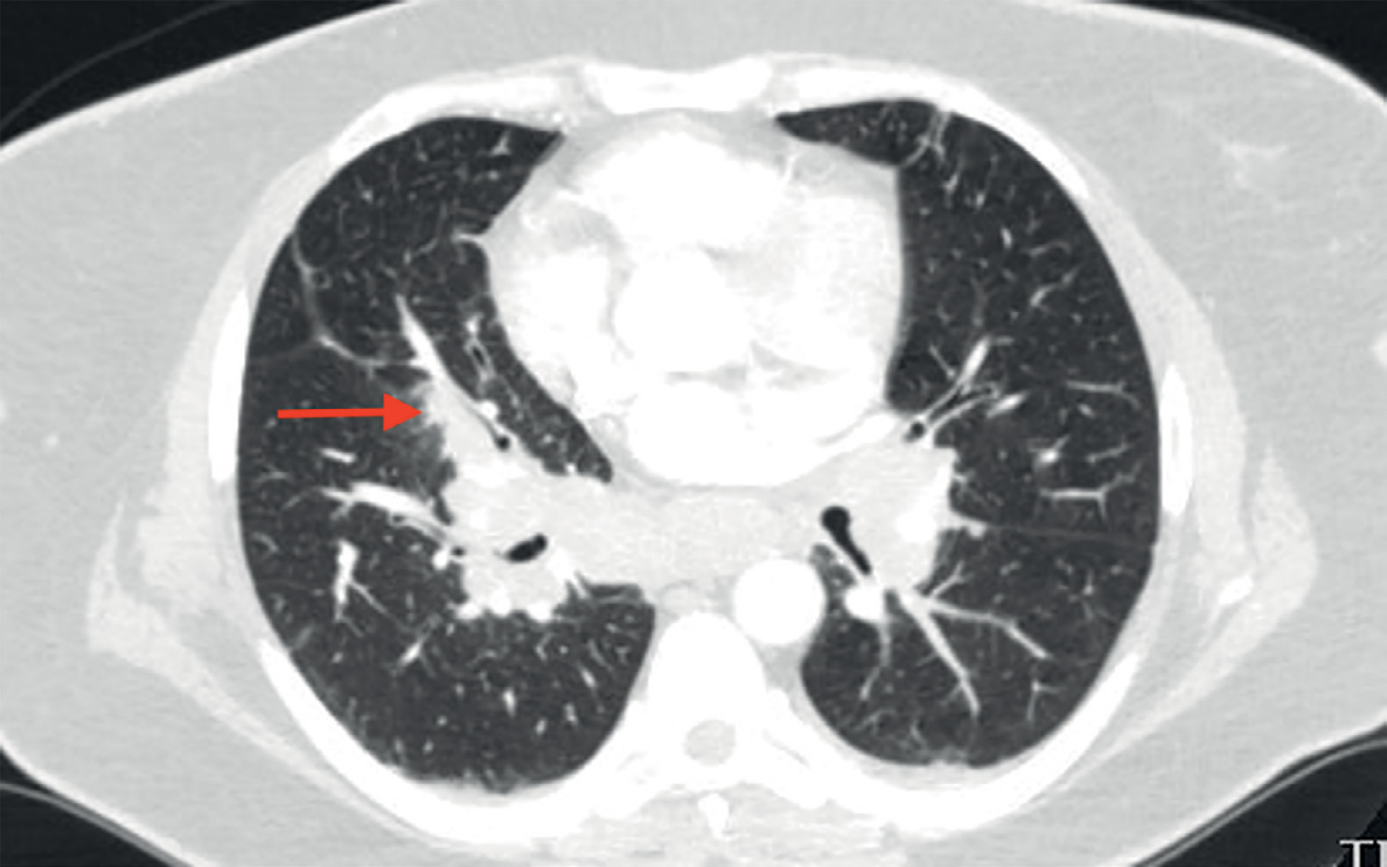
Рисунок 2. Саркоидоз легких и внутригрудных лимфоузлов у пациентки М. Невыраженное уплотнение перибронховаскулярного интерстиция (указано стрелкой).

**Figure:**
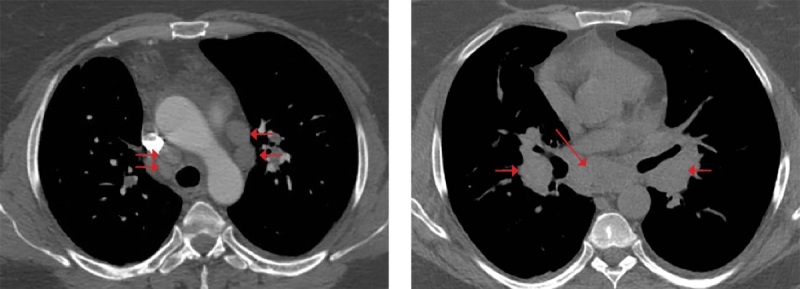
Рисунок 3. Увеличение внутригрудных лимфоузлов как проявление саркоидоза у пациентки М.

Учитывая наличие типичной клинико-рентгенологической симптоматики острого течения саркоидоза и отсутствие данных за другие возможные причины поражения легких, морфологическую верификацию диагноза не проводили. Жалобы на мышечную слабость и тошноту у пациентки после недавней аденомэктомии по поводу болезни Иценко–Кушинга были расценены как декомпенсация надпочечниковой недостаточности вследствие присоединившегося системного воспаления. Доза гидрокортизона увеличена до 25 мг/сут (10 мг утром, 10 мг в обед и 5 мг вечером), на фоне которой тошнота и слабость прекратились. В дальнейшем, в течение нескольких месяцев отмечалось постепенное исчезновение узловатой эритемы. При динамической КТ органов грудной клетки, выполненной через год после диагностики саркоидоза, очаговые тени и увеличение внутригрудных лимфоузлов сохранялись, но не прогрессировали, что косвенно подтвердило диагноз саркоидоза; отмечалась положительная динамика показателей СОЭ и СРБ. Пациентка продолжала прием гидрокортизона в дозе 25 мг/сут. В дальнейшем связь с пациенткой оборвалась в связи со сменой места жительства.

## ОБСУЖДЕНИЕ

Болезнь Иценко–Кушинга характеризуется повышенным уровнем кортизола в крови вследствие синтеза АКТГ опухолью гипофиза, что сопровождается такими же биологическими эффектами, как и терапия системными глюкокортикостероидами. Высокий уровень кортизола способствует развитию иммуносупрессии и, помимо возрастания риска бактериальных инфекций, может маскировать имеющиеся аутоиммунные заболеваний. При успешном хирургическом лечении болезни Иценко–Кушинга наступает быстрое снижение уровня кортизола, что приводит к уменьшению негативных эффектов гиперкортицизма, в том числе ослаблению противовоспалительного и иммуносупрессивного влияния. Это, в свою очередь, создает предпосылки для появления или прогрессирования имеющихся у пациента воспалительных иммунопатологических процессов, в том числе определяющих развитие саркоидоза.

Саркоидоз в настоящее время рассматривается как заболевание неизвестной этиологии, при котором происходят формирование неказеифицирующихся гранулем в различных органах и тканях, активация Т-лимфоцитов, макрофагов и других иммунокомпетентных клеток, что сопровождается повышенным синтезом провоспалительных цитокинов и хемокинов. Наиболее часто выявляют саркоидоз легких и внутригрудных лимфоузлов. Саркоидоз может дебютировать как островоспалительное заболевание, например в виде синдрома Лефгрена (узловатая эритема, артриты, лихорадка, увеличение внутригрудных лимфоузлов).

Системные глюкокортикостероиды, подавляя воспалительную активность заболевания, оказываются эффективными в лечении большинства случаев острого саркоидоза, однако непродолжительный прием этих препаратов ассоциируется с увеличением вероятности его рецидива и формирования хронической прогрессирующей формы болезни, что, наряду с ­известными побочными эффектами стероидов, ограничивает их использование при синдроме Лефгрена [13][14]. Риск рецидива саркоидоза может быть особенно высоким в течение первых месяцев после завершения короткого курса глюкокортикостероидной терапии, что является следствием недостаточного по длительности иммуносупрессивного эффекта лечения, приведшего не к исчезновению, а лишь к уменьшению активности самоиндуцирующегося иммунопатологического процесса. Активизации воспаления может способствовать и рикошетный гипокортицизм, возникший после прекращения терапии системными глюкокортикостероидами. Проводя клинические параллели между экзогенным и эндогенным гипокортицизмом, следует упомянуть о небольшом числе имеющихся на сегодняшний день данных, подтверждающих вышеупомянутую концепцию. Описанные в литературе случаи возникновения или обострения различных аутоиммунных заболеваний (тиреоидита, ревматоидного артрита, воспалительных заболеваний кишечника, системной красной волчанки, серонегативного артрита, ретинального васкулита, болезни Грейвса, склерозирующего панкреатохолангита, атопического дерматита, псориаза, пемфигоида, витилиго, а также саркоидоза [8–12][15–18]) после удаления кортикотропиномы, аденомы надпочечника или эктопической аденомы остаются немногочисленными. Указанные заболевания дебютировали или обострялись в разные сроки (от 1 мес до нескольких лет) после проведенного оперативного лечения, поэтому говорить о причинно-следственной связи во всех случаях затруднительно. Возникновение острых и первично хронических форм саркоидоза, согласно данным, суммированным Noreña-Rengifo B. и соавт. (2019) [19], наиболее часто наблюдалось в течение 5 мес после проведенного лечения, однако в двух случаях эти изменения возникали через 12 и 72 мес.

В представленном нами случае через 4 мес после успешного нейрохирургического лечения болезни Иценко–Кушинга у пациентки появилась симптоматика саркоидоза острого течения (синдром Лефгрена). Терапия гидрокортизоном, которую на тот момент получала пациентка (в дозе 15 мг/сут), не могла предотвратить развитие системного воспаления, так как доза глюкокортикостероидов примерно соответствовала средней суточной потребности в этих гормонах и не обладала иммуносупрессивными свойствами. Стихание симптомов синдрома Лефгрена отмечалось при увеличении дозы гидрокортизона до 25 мг/сут. Такая доза глюкокортикостероидов выше тех доз, которые получают пациенты после оперативного лечения болезни Иценко–Кушинга с заместительной целью. Однако в настоящем случае высокая доза глюкокортикостероидов была обусловлена необходимостью иммуносупрессивной терапии из-за развившегося саркоидоза.

Синдром Лефгрена предполагает наличие симптомокомплекса, включающего узловатую эритему, артриты, повышение температуры и рентгенологические изменения в виде внутригрудной лимфаденопатии [20]. На КТ органов грудной клетки при синдроме Лефгрена возможна визуализация поражения легочной паренхимы вследствие ее вовлечения при саркоидозе. Наряду с характерным для синдрома Лефгрена увеличением внутригрудных лимфоузлов, на КТ легких у нашей пациентки выявлялись интерстициальные изменения в легочной паренхиме (мелкоочаговые и ретикулярные тени), не исчезнувшие в течение более 1 года, что свидетельствует в пользу трансформации острой формы саркоидоза в хроническую [20]. Лабораторные тесты при синдроме Лефгрена выявляют признаки повышения острофазовых показателей воспаления в крови (СОЭ, СРБ). Показатели функции внешнего дыхания, легочной диффузии, результаты тестов толерантности к нагрузке при синдроме Лефгрена, как правило, соответствуют нормальным показателям и не являются методом диагностики заболевания, поэтому эти исследования у данной пациентки не проводились.

## ЗАКЛЮЧЕНИЕ

Данное наблюдение дополняет имеющиеся сведения о крайне редких случаях развития саркоидоза после успешного лечения эндогенного гиперкортицизма. Результаты наблюдения за нашей пациенткой подтверждают концепцию об иммуносупрессивном влиянии высоких концентраций эндогенного кортизола на текущие или формирующиеся аутоиммунные процессы и о повышении вероятности развития системного воспалительного заболевания на фоне снижения уровня кортизола крови до физиологических показателей.

## ДОПОЛНИТЕЛЬНАЯ ИНФОРМАЦИЯ

Источники финансирования. Работа выполнена по инициативе авторов без привлечения финансирования.

Конфликт интересов. Авторы декларируют отсутствие явных и потенциальных конфликтов интересов, связанных с содержанием настоящей статьи.

Участие авторов. Захарова П.А. — вклад автора по критерию 1, по критерию 2; Иловайская И.А. — вклад автора по критерию 1, по критерию 2; Терпигорев С.А. — вклад автора по критерию 1, по критерию 2; Комердус И.В. — вклад автора по критерию 1, по критерию 2; Луговская А.Ю. — вклад автора по критерию 1, по критерию 2.

Все авторы одобрили финальную версию статьи перед публикацией, выразили согласие нести ответственность за все аспекты работы, подразумевающую надлежащее изучение и решение вопросов, связанных с точностью или добросовестностью любой части работы.

Согласие пациента. Пациент добровольно подписал информированное согласие на публикацию персональной медицинской информации в обезличенной форме.
